# Post-Heroin Post-Traumatic Stress Disorder Spectrum: Heroin Addiction as a Generator of Trauma Sensitisation in Everyday Life: A Perspective Review

**DOI:** 10.3390/jcm14186662

**Published:** 2025-09-22

**Authors:** Icro Maremmani, Filippo Della Rocca, Manuel Glauco Carbone, Angelo Giovanni Icro Maremmani

**Affiliations:** 1 VP Dole Research Group, PISA-School of Addiction Medicine, G. De Lisio Institute of Behavioural Sciences, Via di Pratale 3, 56121 Pisa, Italy; icromaremmani@gmail.com (I.M.); filippo.dellarocca@yahoo.it (F.D.R.); manuelglaucocarbone@gmail.com (M.G.C.); 2 Addiction Research Methods Institute, World Federation for the Treatment of Opioid Dependence, 225 Varick Street, Suite 402, New York, NY 10014, USA; 3 UniCamillus, International Medical University in Rome, Via di Sant’Alessandro 8, 00131 Rome, Italy; 4 Addiction Unit, Department of Mental Health and Addictions, ASL5 Liguria NHS, Via Dalmazia 1, 19124 La Spezia, Italy; 5 Division of Psychiatry, Department of Medicine and Surgery, University of Insubria, Viale Luigi Borri 57, 21100 Varese, Italy

**Keywords:** alcohol use disorder, heroin use disorder, sodium oxybate, agonist opioid treatment, long-term treatment, retention in treatment, efficacy, safety

## Abstract

**Background:** Heroin addiction is associated with profound dysregulation of the endogenous opioid and stress response systems, yet current diagnostic frameworks may inadequately capture the traumatising aspects of this condition. This perspective proposes the concept of post-heroin post-traumatic stress spectrum (pH-PTSD/S) as a clinical syndrome emerging from chronic opioid-induced neurobiological and psychosocial dysregulation, even in the absence of Criterion A trauma. **Methods:** The authors review evidence from neuroendocrinology, behavioural neuroscience, and clinical psychopathology to support a sensitisation-based model of trauma vulnerability in heroin use disorder (HUD). **Results:** Findings suggest that HUD patients frequently exhibit PTSD-spectrum symptoms, including hyperarousal, avoidance, emotional dysregulation, and altered stress reactivity. Opioid agonist treatment (OAT) may mitigate these symptoms by stabilising HPA axis function and reducing exposure to trauma-related contexts. The pH-PTSD/S construct, measured through a dedicated instrument, identifies patients with subthreshold trauma-related symptoms and greater psychopathological burden. **Conclusions:** Heroin dependence may constitute a traumatising condition, requiring dimensional diagnostic tools and trauma-informed treatment strategies. Further research is needed to validate the nosological status of pH-PTSD/S, clarify its distinction from protracted withdrawal or complex PTSD, and determine its implications for OAT duration and integrated care pathways.

## 1. Introduction

Post-Traumatic Stress Disorder (PTSD) has traditionally been defined as a clinical response to exposure to life-threatening events, with a symptom cluster encompassing intrusive re-experiencing, avoidance, emotional numbing, and physiological hyperarousal. However, this definition has significantly evolved in recent decades. Current conceptual models have expanded to include not only catastrophic events but also the cumulative effects of chronic interpersonal adversity, socio-environmental stressors, and psychiatric vulnerability, leading to the emergence of broader trauma-spectrum conditions [1,2].

Within this revised framework, we suggest that heroin use disorder (HUD) may itself serve as a traumatising process, independent of any specific traumatic event [3]. The lived experience of heroin dependence—particularly the repeated cycle of intoxication, withdrawal, craving, and survival-driven behaviour—may function as a chronic internal stressor, gradually undermining neurobiological resilience over time.

This leads to the core hypothesis of the present paper: heroin addiction may induce a sustained process of psychological and physiological sensitisation to stress, similar to what is observed in opioid-induced hyperalgesia. Just as repeated withdrawal lowers the threshold for pain sensitivity, repeated episodes of withdrawal and the existential distress associated with heroin use may reduce the threshold for maladaptive stress responses, contributing to a PTSD-like condition without the need for an initial external trauma.

We describe this condition as the Post-Heroin Post-Traumatic Stress Disorder Spectrum (pH-PTSD/S), a clinical syndrome characterised by intrusive symptoms, affective dysregulation, and hyperarousal in individuals with HUD—even without a traditional trauma history.

To support this framework, the following sections will address:

- The bidirectional relationship between PTSD and substance use, including epidemiological links, clinical symptom overlap, and theoretical models.
- From classical PTSD to Opioid-Trauma Syndromes, with an emphasis on neurobiological mechanisms of stress sensitisation.
- Clinical evidence of the post-heroin PTSD spectrum, derived from observational and psychometric data.
- The implications of this model for trauma-informed care, including a discussion on whether opioid agonist treatment (OAT) can reduce PTSD-spectrum symptoms in this population.
- Limitations and Strengths of pH-PTSD/S.

By reconceptualising heroin dependence as both an addictive and traumagenic condition, this paper aims to promote a neurobiologically integrated and clinically actionable understanding of dual disorders involving HUD and trauma-spectrum psychopathology.

## 2. The Bidirectional Link Between PTSD and Substance Use

### 2.1. The Relationship Between PTSD and SUD

#### 2.1.1. Epidemiological Links Between PTSD and SUD

Epidemiological research consistently documents a notable co-occurrence between PTSD and SUD across clinical and community samples. Lifetime PTSD prevalence among treatment-seeking SUD patients is high—typically reported to range from 36% to 50%—a pattern that has been repeatedly observed across various clinical settings [4,5]. Accordingly, a cross-sectional study of a UK SUD inpatient clinical population found that more than one-third of substance-dependent patients met the criteria for current PTSD, and over half met the criteria for lifetime PTSD [6]. Additionally, an analysis of a large sample of African-American substance users who had not received treatment revealed that 40% of respondents reported a history of traumatic experiences. Notably, nearly half of these individuals subsequently developed PTSD [7]. Conversely, community surveys indicate that lifetime SUD affects approximately 20–35% of individuals with PTSD, with large population datasets (e.g., National Epidemiological Survey on Alcohol and Related Conditions—III (NESARC-III)) showing significantly elevated odds for both alcohol- and substance use disorders among people meeting PTSD criteria [5,8].

It is noteworthy that the extent of comorbidity is not consistent; higher co-occurrence is observed among particular groups, notably military veterans [9], women exposed to interpersonal violence [10], and adolescents with early trauma histories [11,12]. Furthermore, research has documented some specificity regarding the type of rewarding substance, as the prevalence of PTSD is higher in drug dependence compared to alcohol dependence [13]. However, gender disparities exist concerning the association between early-onset alcohol use and alcohol dependence, with female subjects showing a greater prevalence of these conditions following exposure to traumatic events [6]. Additionally, a history of abuse has been reported to increase the likelihood of alcohol or substance misuse among women [14]. Conversely, studies have shown that early-onset use of marijuana and heroin, along with alcohol and opioid dependence, are associated with traumatic exposure in male subjects [7].

Notably, the presence of PTSD and SUD together results in a significant combined illness burden. Findings from various studies and systematic reviews have shown a moderate to high level of confidence in the negative impact of comorbidity on prognosis and service requirements. Specifically, patients with both conditions exhibit a higher prevalence of medical comorbidities, increased rates of suicide attempts, a greater incidence of legal issues, and considerably higher utilisation of healthcare services compared to those with a single diagnosis [15,16]. Furthermore, individuals with both PTSD and SUD display more severe symptoms and experience poorer treatment outcomes than those with either disorder alone [17,18]. From a functional perspective, these individuals show increased social and occupational impairment [19], along with reduced retention in SUD treatment and higher relapse rates [20].

#### 2.1.2. Clinical Overlap and Symptom Interactions

PTSD and SUD share multiple symptom domains and can worsen each other, making it hard to tell apart disorder-specific features from interactional ones. A key area involves physiological arousal and withdrawal symptoms. Core PTSD signs, such as sleep issues, hypervigilance, and irritability, tend to get worse during substance withdrawal, leading to clinical profiles that resemble or intensify PTSD hyperarousal. Existing evidence ranges from detailed clinical case reports to neurobiological reviews specific to the disorders. These sources suggest that withdrawal can both mimic and strengthen PTSD arousal symptoms [5,21,22]. Another overlapping area includes cue-induced craving and trauma reminders. It has been repeatedly shown that trauma cues and drug-related cues can trigger similar conditioned emotional and motivational responses. Preclinical models indicate that previous stress or trauma increases drug-seeking behaviour—such as stress-triggered methamphetamine seeking—and clinical reports show similar patterns, giving moderate evidence for stimulus generalisation and cue-reactivity across disorders [23,24,25]. A further shared feature is the complexity of treatment and its effects on outcomes. Rigorous systematic reviews have shown that integrated treatments targeting both PTSD and SUD tend to produce better outcomes for PTSD symptoms than sequential or addiction-only approaches. However, substance use outcomes often face greater challenges and complexity when it comes to changing them [26].

The clinical literature, when considered collectively, supports a reciprocal symptom-amplification model where PTSD and SUD interact dynamically. This model proposes that symptom domains in one disorder can be intensified by neurobiological and behavioural processes in the other, making diagnosis and management more complex. However, some findings indicate that PTSD is an independent risk factor for poorer outcomes in SUD [13]. In this context, it has been hypothesised that the increased severity of SUD in patients with PTSD is not directly caused by PTSD itself, but instead results from the frequent co-occurrence of PTSD and related psychopathologies [27].

Overall, longitudinal syntheses and comprehensive reviews show a largely bidirectional relationship, where PTSD often comes before the development of SUD. At the same time, significant or extended substance use can, in turn, increase vulnerability to subsequent PTSD after new traumatic exposures [28].

#### 2.1.3. Etiological Frameworks

Multiple, not mutually exclusive, etiological models have been proposed to explain the frequent co-occurrence of PTSD and SUD. Each model is supported to varying degrees by epidemiological, clinical, and mechanistic evidence.

The Self-Medication Hypothesis (SMH) proposes that individuals might use substances to cope with PTSD symptoms. This theory suggests that traumatic stress causes negative emotional states, which people may attempt to manage by using drugs or alcohol. While this behaviour can offer temporary relief, it often leads to physical dependence and withdrawal, potentially exacerbating PTSD-like symptoms.

The SMH, originally developed by Khantzian, suggests that individuals see drug use as the most effective way to relieve psychological distress and suffering. This view describes drug use as a “drive to use,” reflecting the motivations behind self-medication behaviours [29]. Additionally, physiological arousal during withdrawal from dependence-inducing substances may worsen PTSD symptoms, thereby increasing the risk of relapse and perpetuating a maladaptive cycle of self-medication and dysfunction [30].

Symptoms commonly associated with this condition include anxiety, hyperarousal, impulsivity linked to increased stress reactivity, and memory difficulties. Patients may also exhibit heightened emotional responses to trauma-related cues and intrusive re-experiencing of traumatic memories, especially within the context of conditioned fear responses [31,32]. Within the SMH, a strong interaction is suggested between a drug’s pharmacological effects and the individual’s psychopathology, further complicated by underlying vulnerability traits. Consequently, psychotropic substances—including illegal drugs, prescribed medications, and alcohol—may be used by PTSD sufferers to cope with stress, temporarily reduce psychological burden, and enhance impulse control [5].

The SMH also encompasses the idea that substance choice is not random, but rather related to the specific type of distress experienced. For example, adolescents with dysregulated stress reactivity might try to modulate their arousal levels through substance use—either to reduce hyperarousal or to increase hypoarousal states [33]. Clinical evidence indicates that the type of substance (e.g., CNS depressants versus stimulants) may be chosen based on the dominant PTSD symptom profile. Patients with PTSD who develop alcohol dependence, for instance, often exhibit more intense arousal symptoms than those with co-occurring cocaine dependence [34].

A substantial body of literature supports a directional pathway in which PTSD precedes substance use or dependence. Preclinical studies indicate that PTSD-related dysregulation of the corticotropin-releasing factor (CRF) and noradrenergic systems may gradually heighten stress responses. Patients may therefore self-medicate with sedatives, hypnotics, or alcohol to break this feedback loop or to alleviate distress linked to trauma reminders [35].

While PTSD is frequently comorbid with other psychiatric disorders, SUD is among the most common and clinically significant. Comorbidity between PTSD and SUD complicates clinical outcomes for both conditions [17,18]. A population-based study of PTSD patients showed that individuals with a history of substance use—particularly involving cocaine and cannabis—had notably higher PTSD symptom severity compared to non-users [36].

In summary, the SMH is well supported by empirical evidence, particularly concerning alcohol use. Its significance has been extensively discussed in comprehensive reviews and research centred on opioids. However, the evidence relating to opioids remains moderate due to a lack of prospective, longitudinal studies [4,5,28].

The Shared-Vulnerability Model offers an alternative explanation, highlighting complex, convergent vulnerability factors that heighten the risk of both PTSD and SUD. According to this perspective, shared biological, genetic, and environmental mechanisms may account for the concurrent development of the two disorders.

Stress circuit dysregulation has been observed in both PTSD and SUD. Dysregulation of the Hypothalamic–Pituitary–Adrenal (HPA) axis contributes to altered stress responses, as documented in both clinical and preclinical studies [23,37]. Animal models have demonstrated sustained elevations in corticosterone following trauma exposure, which may increase vulnerability to later drug-seeking behaviours [25].

Neurobiological maladaptations in shared reward and fear regulation systems have also been associated with both disorders. Changes in mesolimbic dopamine, glutamatergic, GABAergic, and noradrenergic pathways have been observed. One key hypothesis proposes that upregulation of the dynorphin/κ-opioid receptor (DYN/KOR) system serves as a common mechanism underlying both negative affect and drug craving [24,38].

In summary, the Shared-Vulnerability Model indicates that PTSD and SUD may arise from common vulnerability factors affecting core neurobiological systems. Genetic, neuroendocrine, and environmental influences influence brain function, shape behavioural traits, and collectively contribute to stress sensitivity and substance misuse. Elevated catecholamine levels, disrupted HPA-axis functioning, and widespread neurochemical as well as structural changes in brain regions responsible for executive control and decision-making might establish a shared neurobiological basis for both disorders [39].

The Traumagenic Hypothesis presents an alternative explanation, suggesting that early-life trauma and adverse developmental conditions can lead to enduring neurobiological changes that increase vulnerability to both PTSD and SUD. According to this hypothesis, stress-related disruptions in neurotransmitter systems may influence not only trauma-related psychopathology but also a greater likelihood of substance experimentation, alcohol use, and eventual dependency [31,40]. Neuroimaging studies have demonstrated that early adversity can alter the structure and function of frontoparietal and salience networks, thereby elevating the risk for SUD later in life [11,12].

These findings support developmental models of bidirectional vulnerability, which propose that early stress exposure affects neural circuitry in ways that predispose individuals to both PTSD and substance-related disorders. This maladaptive neurodevelopmental pathway encourages behavioural disinhibition and risk-taking tendencies, heightening susceptibility throughout life. Furthermore, childhood adversity has consistently been associated with a higher likelihood of engaging in high-risk behaviours, developing substance use disorders, experiencing additional trauma in adulthood, and ultimately meeting the diagnostic criteria for PTSD [31,40].

Taken together, these observations highlight the vital role of developmental trauma in the co-occurrence and mutual reinforcement of PTSD and SUD. The interaction between early-life adversity, neurobiological changes, and behavioural vulnerability underlines the importance of trauma-informed approaches in prevention and treatment strategies for dual diagnoses.

### 2.2. The “Addiction-Induced PTSD Vulnerability” Model: SUD as an Independent Risk Factor in Increasing Individual Susceptibility to PTSD

The Addiction-Induced PTSD Vulnerability Model further advances this discussion by proposing that substance use disorders (SUDs) may act as independent risk factors that heighten vulnerability to PTSD. While extensive research has examined how prolonged stress and trauma exposure contribute to the development [3,6,41], emerging evidence suggests that the opposite relationship—where substance use increases vulnerability to trauma—deserves equal attention. Several studies show that substance use significantly affects stress systems and response mechanisms, even in individuals without preexisting psychiatric conditions [42,43,44,45,46].

Physiologically, individuals with SUD often show increased baseline activity of brain stress systems, as evidenced by higher adrenocorticotropic hormone (ACTH) and hair cortisol levels compared to healthy controls [47,48,49,50]. Epidemiologically, substance-abusing individuals frequently expose themselves to high-risk environments that raise their exposure to trauma [51,52,53]. Clinically, chronic substance use has been associated with increased arousal and anxiety, greater emotional dysregulation, and impaired coping mechanisms, all of which may increase vulnerability to post-traumatic psychopathology [3,54,55,56].

Notably, individuals with SUDs have a higher likelihood of developing PTSD after trauma exposure compared to non-users, due to greater trauma exposure and increased stress reactivity. This was demonstrated in a study of individuals with cocaine use disorder, where trauma sustained during drug procurement resulted in subsequent PTSD [57]. Furthermore, emotion-focused coping strategies—common among those with co-occurring PTSD and SUD—seem to mediate the link between trauma exposure and substance use, leading to poorer treatment outcomes [58].

On a neurobiological level, dysregulation of the hypothalamic–pituitary–adrenal (HPA) axis has been consistently observed across various substances, including alcohol, opioids, cocaine, nicotine, and cannabinoids [44,59,60,61,62]. Chronic substance use disrupts stress and reward systems, particularly the corticotropin-releasing factor (CRF) and dopaminergic pathways, thereby facilitating maladaptive neuroadaptations. These disruptions are phase-dependent: acute intake often results in HPA hyperactivation (except in opioids), while chronic use leads to blunted responses, and withdrawal induces hyperresponsiveness, increasing relapse risk [63,64,65,66,67,68].

Animal and human studies have shown that both stress and addictive substances activate overlapping neural circuits, including the dopaminergic mesolimbic pathway and the extended amygdala, reinforcing their bidirectional interaction [69,70,71,72,73]. Importantly, these shared circuits may form the basis of common vulnerability mechanisms and help explain the frequent co-occurrence of PTSD and SUD.

Overall, dysregulation of the brain’s stress systems is not just a consequence but a key driver of addiction-related processes. This framework supports the hypothesis that SUD may occur before and enhance the development of PTSD by causing persistent neurobiological changes—particularly within the CRF and noradrenergic systems—that reduce resilience to future trauma [5,28].

## 3. From Classical PTSD to Opioid-Trauma Syndromes

### 3.1. Rethinking PTSD: From Diagnostic Criteria to Trauma Spectrum

In recent years, the traditional view of PTSD as a separate diagnostic entity has been increasingly challenged by a more nuanced, dimensional perspective. While classical PTSD remains tied to specific diagnostic criteria and the experience of life-threatening events, clinical research has highlighted a broader PTSD spectrum that includes symptom constellations arising from chronic adversity, sustained social stress, or cumulative trauma exposures [2,74]. This spectrum-based model accounts for the diversity and gradation of trauma-related phenomena, such as subthreshold PTSD, complex PTSD, and persistent emotional dysregulation rooted in long-term interpersonal or environmental adversity.

Critically, this reconceptualisation has important implications for clinical assessment and intervention. Many individuals experiencing debilitating trauma-related symptoms do not meet the strict criteria for a PTSD diagnosis, yet they exhibit clear signs of emotional, cognitive, and physiological dysregulation. Such cases are particularly common among populations exposed to ongoing low-level threats or structurally embedded stressors. Chronic substance dependence, social marginalisation, and exposure to community violence have all been recognised as environmental factors capable of producing traumatised phenotypes through mechanisms similar to those found in classical trauma models.

The impact of prolonged social marginalisation has been confirmed by cross-national longitudinal data. Young adults facing unstable housing, judicial involvement, unemployment, or minority status show higher rates of psychological distress and substance use, along with distinctive patterns of emotional dysregulation and relational dysfunction. These outcomes align with trauma-spectrum presentations and appear to arise not from isolated incidents, but from ongoing exposure to exclusion and structural vulnerability [75].

Further evidence from the field of health disparities supports this view. Structural racism, discrimination, and exclusion serve as chronic stressors with significant psychobiological effects. Research has shown that racial discrimination can dysregulate the HPA axis through anxiety-related pathways [76], increase pro-inflammatory biomarkers over time—even after controlling for standard health risks [77]—and alter both peripheral and central neurophysiological systems in ways similar to PTSD symptoms [78]. Social isolation, as another aspect of marginalisation, has also been linked to neurobiological adaptations commonly observed in PTSD. These include disruption of the HPA axis, impaired immune signalling, changes in neural plasticity within limbic and prefrontal regions, and even telomere shortening, suggesting an acceleration of biological ageing [79]. Overall, these mechanisms demonstrate how social isolation functions not only as a stressor but also as a traumagenic condition capable of producing long-lasting psychological and physical effects.

Similarly, exposure to community violence—particularly in socioeconomically disadvantaged environments—has become a major factor in trauma-related symptoms. Both direct victimisation and witnessing violence are associated with PTSD-like symptoms, which may be subclinical but can still affect daily functioning. Neuroimaging studies confirm that these experiences alter brain connectivity within key emotional and regulatory networks [80,81]. These findings support cumulative threat models, which propose that repeated violence activates biological systems involved in stress, emotion, and immune regulation, ultimately contributing to long-term physical and mental health issues [82].

Importantly, these traumagenic contexts are not merely external to addiction; they are arguably fundamental to the heroin-using experience. Individuals with HUD often reside in environments shaped by social exclusion, community violence, and interpersonal instability. Heroin dependence, in this context, develops not only as a response to past trauma but also functions as a chronic traumatising condition in its own right. The lifestyle linked to heroin use—characterised by cycles of withdrawal, criminalisation, precarious survival, and emotional desensitisation—exposes individuals to repeated stressors that can trigger and sustain trauma-spectrum symptoms. From this perspective, heroin addiction should be regarded not simply as a comorbid condition of PTSD but as a source of trauma sensitisation—one capable of producing a PTSD/S phenotype.

### 3.2. Opioid-Induced Stress Dysregulation and Neurobiological Trauma

On a neurobiological level, a complex interaction exists between the endogenous opioid system, its dysregulation in cases of HUD, and the brain’s stress systems. This interaction may explain the observed link between HUD and a heightened risk of subsequent PTSD. To clarify, the hypothesis that HUD increases vulnerability to PTSD through disruptions in the endogenous stress regulatory systems has been proposed [28,30].

The regulation of the hypothalamic–pituitary–adrenal (HPA) axis by the endogenous opioid system happens through three main mechanisms, each helping to modulate stress responses [83]. First, β-endorphin neurons originating in the arcuate nucleus have a direct inhibitory effect on corticotropin-releasing factor (CRF) neurons within the hypothalamus, thus reducing the release of adrenocorticotropic hormone (ACTH) [84]. Second, endogenous opioids decrease noradrenaline release from the locus coeruleus, mainly through their action on opioid-sensitive neurons located in the brainstem [85,86]. Third, an additional layer of inhibition seems to be mediated indirectly: endogenous opioids suppress the activity of norepinephrine neurons that would otherwise stimulate CRF, thereby lowering CRF output via a secondary regulatory pathway [86].

Therefore, using opioid antagonists to block the endogenous opioid system has been shown to cause disinhibition of opioid regulation of the HPA axis. This results in increased HPA activity, as evidenced by an immediate rise in ACTH and cortisol levels [87,88]. Furthermore, opioid antagonists such as intravenous naloxone or oral naltrexone have been utilised to assess the functional status of hypothalamic opioid tone [89,90]. As a consequence, a decrease in endogenous opioid activity generally leads to disinhibition of stress hormones.

Interestingly, endogenous opioids, which are usually released in response to stress, have been shown to contribute to stress-induced analgesia and may be mobilised as a compensatory mechanism in PTSD [30,91,92]. Accordingly, research consistently indicates that patients with PTSD exhibit an exaggerated HPA axis response to the administration of naloxone. Naloxone has also demonstrated efficacy in reversing the analgesia experienced by PTSD patients following exposure to traumatic reminders. Furthermore, patients with PTSD have been observed to show increased cerebrospinal fluid β-endorphin levels, suggesting that the endogenous opioid system may be more active in these individuals. Notably, naltrexone has proven effective in managing symptoms related to dissociation and flashbacks in trauma-experienced patients [93,94]. Consequently, the downregulation of endogenous opioid signalling in HUD may lead to impaired homeostatic stress regulation, which could increase susceptibility to PTSD [28,30].

Furthermore, administering exogenous opioids disrupts this balance by inhibiting glucocorticoid release, thereby removing a crucial negative feedback mechanism within the central stress response [44,54,65,95,96]. While giving short-acting opioids shortly after potentially traumatic events has been shown to reduce the risk of developing PTSD symptoms [97,98], opioid-induced suppression of glucocorticoids may be linked to the consolidation of traumatic memories. Specifically, as the stress-buffering effects of endogenous opioids decline, the individual’s ability to regulate stress becomes destabilised, increasing vulnerability to trauma-related symptoms. Preclinical studies also show that chronic opioid pretreatment can enhance fear learning. For example, animal models using Stress-Enhanced Fear Learning (SEFL) demonstrate that prior opioid exposure strengthens associative fear memories, which can persist long after stopping drug use. This suggests that opioid-induced neural changes in areas such as the basolateral amygdala may make individuals more susceptible to future stressors [99]. Overall, this indicates that individuals using short-acting opioids might experience greater stress when exposed to new stressors [28]. Simultaneously, long-term opioid administration leads to tolerance within the locus coeruleus, disrupting the balance between opioidergic activity and CRF. In particular, tolerance reduces opioid-mediated inhibition of the locus coeruleus, leaving CRF activity relatively unrestrained. This creates a feed-forward loop that amplifies stress responses. Such an imbalance not only intensifies stress but also promotes drug-seeking behaviour as individuals try to compensate for reduced endogenous opioid function [86,100]. Additionally, opioid κ receptor (KOR) signalling appears to mediate stress susceptibility. Activation of this system under chronic stress may result in anxiety-like and dysphoric states. Conversely, KOR antagonists have been shown to reverse these effects, indicating that opioid systems directly influence the neurobiology of trauma sensitisation [101].

Finally, HUD has been shown to cause significant dysregulation of the stress system, particularly marked by hyperactivation of the HPA axis, leading to exaggerated cortisol and ACTH responses during both naloxone-precipitated and natural withdrawal [96,102,103,104,105]. This increased reactivity has been proven to raise stress vulnerability, anxiety, and the risk of relapse [3,95,106], with CRF activity elevated during withdrawal and remaining high afterwards [107]. Notably, heightened cortisol and ACTH responses to stress have been observed in individuals with heroin dependence, persisting throughout abstinence [65,108]. Additionally, region-specific CRF changes have been shown to contribute to stress dysregulation. Chronic opioid use has been demonstrated to suppress hypothalamic CRF release [109,110], while simultaneously increasing CRF activity in the amygdala and bed nucleus of the stria terminalis [111,112,113]. These adaptations appear to heighten relapse vulnerability, as evidenced by the reinstatement of drug-seeking behaviour following CRF administration, while CRF antagonists have been found to lessen stress-induced relapse [114,115]. Clinically, opioid dependence has been linked to elevated basal cortisol levels, confirmed through studies analysing both plasma and hair samples [49,116]. This association is accompanied by HPA feedback desensitisation [102,117]. As indicated by Stimmel and Kreek [54], persistent neurobiological changes underpin ongoing relapse vulnerability despite abstinence.

**Figure 1:**
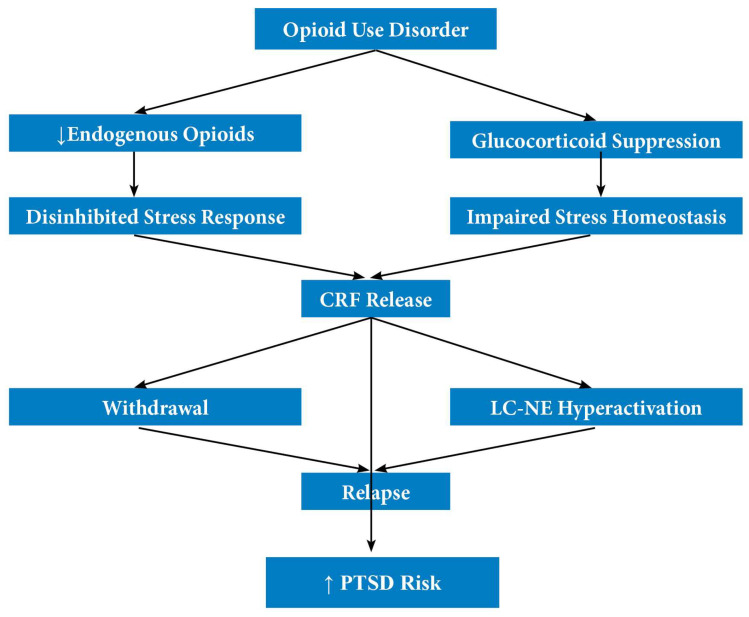
Opioid Use Disorder, Stress System and PTSD Risk.

In summary, HUD has been shown to induce changes in the stress system, resulting in the suppression of glucocorticoids and an increase in CRF and norepinephrine activity. This impairs stress adaptation and extends hyperactivation of central stress circuits. Such dysregulation may promote both relapse and the development of PTSD after trauma that might otherwise resolve without lasting psychopathology. Therefore, these converging lines of evidence suggest that heroin use does not merely correlate with trauma symptoms through environmental factors but actively alters neurobiological systems involved in stress, fear conditioning, and emotional regulation. In this context, heroin dependence also seems to be a neurobiologically traumatising condition, supporting the view that PTSD/S are rooted both in personal experience and in the pharmacological effects of opioids on the traumatisation process (Figure 1).

## 4. Clinical Evidence of the Post-Heroin PTSD Spectrum

This dimensional approach to trauma has become increasingly prominent, particularly in fields such as affective neuroscience and complex trauma research, where symptoms like emotional numbing, interpersonal detachment, and affective dysregulation are recognised as trauma-related, even without a single catastrophic trigger [74,118].

In the field of SUDs, particularly concerning HUD, this trauma-spectrum model is especially significant. Here, the order may be reversed: instead of trauma resulting in substance use, it is the substance use itself, through its physiological, relational, and existential effects, that can foster trauma-like vulnerability. HUD, notably, demonstrates a persistent pattern of threat, loss, and disintegration that closely resembles prolonged traumatic exposure [3].

We therefore propose the construct of pH-PTSD/S: a condition in which chronic heroin dependence results in lasting hypersensitivity to stress and emotional instability, presenting as trauma-spectrum symptoms that endure even after detoxification.

In current addiction discourse, trauma is mainly viewed as a factor that predisposes individuals to substance use. However, growing clinical evidence and theoretical considerations highlight the need to explore the opposite: the possibility that heroin dependence itself can generate a spectrum of post-traumatic stress. We introduce the concept of PTSD/S—a trauma-like response pattern that develops not from a single catastrophic event, but from the prolonged psychological effects of heroin addiction and the increased reactivity to everyday stressors that follow.

Unlike traditional PTSD, which results from sudden, life-threatening events, PTSD/S reflects a long-term sensitisation of the stress response system. People with HUD often exhibit extreme emotional sensitivity even to relatively minor interpersonal conflicts, experiences of loss, or daily frustrations. This phenomenon is not merely psychological fragility; it stems from neurobiological and experiential conditioning through repeated cycles of intoxication, withdrawal, isolation, and existential instability.

Heroin impairs the ability to recover emotionally. Repeated exposure to distressing physical states, such as withdrawal crises, mimics patterns of uncontrollable threats that disrupt the brain’s regulatory systems. Over time, the individual becomes hypersensitive to any negative emotional trigger, including common life events like bereavement, rejection, or work-related stress. These experiences are no longer processed with suitable emotional responses but instead trigger intrusive distress, avoidance, and dysphoric arousal: the key characteristics of post-traumatic syndromes.

### 4.1. Comparative Analysis of Stress Reactivity Before and After Heroin Addiction Onset

A significant contribution to understanding trauma-related processes in heroin addiction comes from a study by the Vincent P. Dole Research Group at the Santa Chiara University Hospital in Pisa, Italy. This research explored the role of life events—including losses and potentially traumatic experiences—in the onset and development of HUD, focusing on the emotional responses they elicited before and after the start of continuous heroin use [119].

The study involved 82 heroin-dependent individuals recruited from addiction services in Central-Northern Italy. Using the Drug Addiction History Questionnaire (DAH-Q) and the Trauma and Loss Spectrum (TALS) instruments, researchers examined the occurrence of significant life events, their emotional impact, and trauma-related reactions, such as avoidance, arousal, and maladaptive behaviours, across two life phases: pre-addiction and addiction.

The findings showed that stressful events were common before starting heroin use, with only a small minority reporting no previous exposure to loss or trauma. However, despite the shorter duration of the addiction phase, the number of reported events increased after heroin use began, indicating that addiction itself encourages exposure to traumatic circumstances, especially legal issues, interpersonal ruptures, and ongoing instability. More importantly, participants reported a significant rise in emotional reactivity after heroin initiation. Reactions to loss events grew stronger in 50% of cases, as did the recall of the most serious event (41.5%), emotional numbing (46.3%), maladaptive behaviours (56.1%), and arousal (42.7%). These changes happened without notable shifts in personality traits, suggesting that the drug-using experience itself heightens trauma responsiveness in ways similar to a post-traumatic stress phenotype.

These results reinforce broader findings in the literature linking SUDs to higher stress sensitivity and reduced capacity for emotional regulation [3,120,121]. This study provides strong evidence that heroin addiction does not simply coexist with trauma but actively helps perpetuate and internalise it, leading to a range of responses that are clinically and phenomenologically consistent with PTSD.

Importantly, while traditional trauma predictors—such as the death of a parent, parental divorce, and emotional neglect—were dominant before the onset of heroin use [3], the addiction phase introduced new categories of traumatic stressors, particularly those associated with criminalisation, arrests, and judicial exposure, as well as repeated interpersonal disruptions. This pattern underscores heroin addiction as both a consequence and a catalyst of traumatisation, consistent with the emerging concept of a pH-PTSD/S. These findings support the view that heroin addiction may condition the individual not only to develop dependency but also to gradually internalise trauma through repeated exposure, emotional dysregulation, and neurobiological sensitisation. Such data provide a robust empirical basis for recognising trauma-spectrum conditions as intrinsic to the lived experience of opioid dependence.

### 4.2. Converging Trajectories: Towards a Unified Model of PTSD and HUD

The growing recognition of trauma-related psychopathology as a spectrum has opened new conceptual pathways for understanding the complex connection between PTSD and SUDs. Moving beyond dual-disorder models, a dimensional approach allows us to see these disorders not as separate but as interconnected and mutually reinforcing processes. In this context, heroin addiction—when viewed through the lens of traumatic sensitisation and emotional dysregulation—may exemplify the PTSD-SUD spectrum.

One of our studies aimed to empirically test this hypothesis by examining the correlations between the severity of heroin addiction and PTSD/S symptoms in a clinical sample of patients undergoing treatment with opioid agonist therapy (OAT) in three Italian addiction units [122]. Data were collected using standardised instruments (DAH-Q; TALS) and analysed to explore the relationship between addiction duration, age of onset, symptom burden, and pharmacological treatment.

Results indicated that the severity of PTSD/S symptoms—particularly arousal, maladaptive coping, and re-experiencing—was positively correlated with the duration of heroin use. Specifically, longer dependence periods and earlier initiation of heroin consumption were linked to more severe trauma-related symptoms. Conversely, patients on higher doses of opioid agonists (e.g., methadone or buprenorphine) reported significantly fewer arousal symptoms, supporting the notion that stable agonist treatment may serve a protective role against trauma responses.

Further analyses confirmed that polysubstance users and patients in more advanced stages of addiction exhibited higher scores across nearly all trauma-related domains, including loss, re-experiencing, avoidance, and emotional dysregulation. Patients with a treatment history also showed higher PTSD/S symptomatology compared to those in their initial therapeutic contact, suggesting cumulative sensitisation to trauma throughout the course of addiction. Although some discriminant functions did not reach statistical significance, the clinical pattern remained consistent.

Importantly, these findings support previous research indicating that short-acting opioids like heroin interfere with the endogenous opioid system, increasing susceptibility to stress and decreasing emotional resilience [3,55]. Conversely, long-acting opioids such as methadone or buprenorphine, when administered in appropriate therapeutic doses, appear to normalise stress responses and reduce trauma-related reactivity. This pharmacological impact aligns with evidence from military and paediatric populations, where early use of opioid analgesics was associated with a lower risk of developing PTSD after acute trauma [97,98]. In our clinical cohort, these effects are demonstrated by the inverse correlation between agonist dose and PTSD/S, particularly in the hyperarousal domain. This supports the notion that agonist therapy not only stabilises addictive behaviour but also aids in emotional regulation and trauma containment.

The broader implication of these findings challenges simple yes-or-no thinking. Instead of seeing PTSD and heroin addiction as separate diagnoses that sometimes overlap, this evidence supports a unified model based on shared vulnerabilities, common neurobiological roots, and mutually strengthening progressions. Traits such as impulsivity, emotional dysregulation, and low resilience, often viewed as individual predispositions, could act as transdiagnostic risk factors affecting both disorders [123].

In summary, our study emphasises that PTSD/S symptoms should be regarded as inherent elements of heroin addiction psychopathology, rather than merely as precursors or co-occurring conditions. The robustness and consistency of the observed correlations imply that HUD both reflects and intensifies traumatised functioning. This realisation calls for a redefinition of clinical strategies—advocating for integrated, trauma-informed approaches instead of isolated treatment pathways—and aligns with the emerging view of PTSD/S as a distinct clinical entity.

### 4.3. Trauma Without Disaster: PTSD/Symptomatology in Heroin Users Compared to Earthquake Survivors

This comparative study aimed to determine whether individuals with HUD, despite never experiencing catastrophic events, display emotional and behavioural responses to loss and trauma similar to those seen in survivors of natural disasters diagnosed with PTSD. Three demographically matched groups were assessed: patients with chronic HUD (N = 77), survivors of the 2009 L’Aquila earthquake (EQ) with diagnosed PTSD (EQ-PTSD, N = 77), and survivors of the same event without PTSD diagnosis (EQ-NPTSD, N = 77). All participants completed standardised instruments, including the SCID-I (Structured Clinical Interview for DSM Disorders) for diagnosing PTSD and the TALS [124].

Despite differences in age and socioeconomic background between the groups, HUD patients reported levels of PTSD/S symptomatology similar to those observed in the EQ-PTSD group and significantly higher than those in the EQ-NPTSD group. Specifically, HUD patients exhibited elevated scores in domains such as grief reactions, hyperarousal, re-experiencing, avoidance, and maladaptive coping. While their symptom levels were slightly lower than those of the EQ-PTSD group, they were consistently and significantly higher than those of the non-PTSD earthquake survivors. Discriminant function analysis confirmed that the HUD group could not be statistically distinguished from the EQ-PTSD group based on emotional reactions to traumatic and loss-related events [125]. This finding supports the hypothesis that chronic heroin addiction may function as a cumulative traumatic condition, capable of inducing psychological alterations typically associated with PTSD. The persistent nature of heroin dependence—marked by withdrawal crises, criminalisation, social marginalisation, and emotional desensitisation—may serve as a continuous stressor, contributing to the emergence of a traumatised phenotype.

The results also align with previous research on other chronic health conditions, such as cancer [126,127,128,129,130,131] and HIV (Human Immunodeficiency Virus) infection [132], where ongoing stress exposure has been shown to trigger PTSD-like symptoms. This body of evidence indicates that the severity of trauma-related symptoms is positively associated with the duration of the stressor and suggests long-term disruption of neurobiological systems, including the endogenous opioid system [96,133,134,135,136,137,138,139,140]. Specifically, stress-triggered activation of the dynorphin/kappa-opioid receptor system may be crucial in the maladaptive and pro-addictive effects of chronic stress, as demonstrated in earlier studies [101,141,142]. This neurobiological framework might also explain the higher rates of maladaptive coping behaviours observed in HUD patients, compared to earthquake survivors who experienced a single acute traumatic event.

Moreover, consistent with earlier work [122,126], the severity of PTSD/S symptoms appears to correlate positively with both the duration and intensity of stress exposure. Our previous study on HUD patients identified more pronounced PTSD/S symptoms in individuals with longer heroin dependence. Similarly, among cancer survivors, earlier disease onset was linked to more severe trauma-related symptoms. Collectively, these findings suggest that PTSD/S severity could serve as a clinical marker of HUD severity, with potential implications for treatment planning. The inverse relationship between agonist opioid medication dosage and arousal symptoms observed in earlier research [122] supports the use of long-acting opioid therapy to regulate stress reactivity. Past studies have demonstrated HPA axis normalisation with extended methadone maintenance [143,144], and other research has noted a protective effect of early opioid analgesic use against PTSD development in acutely traumatised groups [97,145,146]. Finally, this study highlights the role of the opioid system in the pathophysiology of PTSD/S disorders and reinforces the proposal—supported by recent publications from our group—that PTSD/S symptoms should be regarded as a core element of addiction psychopathology [122]. Assessing such symptoms may also serve as a prognostic indicator of recovery potential in HUD patients undergoing agonist opioid treatment.

### 4.4. Trait Psychopathology and Emotional Reactivity

In one of our studies, patients were divided into two subgroups based on the severity of their PTSD/S symptoms, as determined by a TALS total score above or below the cut-off value of 32. The high PTSD/S subgroup (H-PTSD/S) exhibited more severe psychopathology across all five Symptomatologic Checklist (SCL-90)-derived dimensions previously identified as characteristic of SUDs: worthlessness–being trapped (W/BT), somatic symptoms (SSs), sensitivity–psychoticism (S/P), panic–anxiety (PA), and violence–suicide (V/S) [147]. Canonical correlation analysis revealed two distinct but interconnected dimensions: the first, characterised by PA and emotional, physical, and cognitive responses to loss and trauma; and the second, marked by W/BT, V/S, grief reactions, re-experiencing and numbing, arousal symptoms, and personality traits associated with vulnerability to stress [148].

Although clear correlations exist between emotional responses and specific dimensions of psychopathology, no qualitative differences in typology appeared between the L-PTSD/S and H-PTSD/S subgroups. In other words, while symptom severity varied considerably, the fundamental structure of psychopathology remained stable, thus supporting the hypothesis that it is trait-dependent rather than state-dependent, specific to HUD. These findings reinforce prior evidence of a five-factor structure across substances and contexts [149,150,151].

Furthermore, as the PTSD spectrum appears sensitive to addiction progression, its monitoring could act as a prognostic marker in clinical practice. Patients with heightened PTSD/S symptoms—especially those linked to arousal and aggression—might require customised interventions, including stabilising stress systems through OAT [3,152]. The normalisation of HPA-axis function and emotional reactivity via long-term agonist treatment (e.g., methadone or buprenorphine–naloxone) has been documented in previous studies [153,154] and may offer a pathway to functional recovery that surpasses mere abstinence.

### 4.5. Clinical Profiles of PTSD/S Patients

In this study, we aimed to examine the distinct psychopathological and clinical profiles of HUD patients who, during treatment, exhibited a PTSD/S condition (H-PTSD/S) compared with those who did not (L-PTSD/S) [155]. Patients were assessed using standardised instruments to explore differences in illness trajectory, psychopathology, and craving-related behaviours during OAT.

Data were gathered through a demographic survey and the DAH-Q [156], which explored health, social, legal, and clinical factors. Psychopathology was assessed using the SCL-90 [147,157], identifying five dimensions: W/BT, SS, S/P, PA, and V/S. Craving-related behaviours were documented with Crav-Hero [158], which categorised behavioural expressions into six dimensions, including risk-related, cue-induced, and reward/relief-obsessive craving behaviours. The TALS instrument evaluated trauma-related symptoms [125,159].

The presence of Benzodiazepine (BDZ) use and heightened psychopathology were key indicators of H-PTSD/S status, suggesting these factors may indicate treatment resistance or incomplete response to OAT. These findings align with earlier studies linking SUD and PTSD spectrum symptoms [122,125]. While most drug-related behaviours were suppressed during OAT, some residual stress-induced behaviours continued in the H-PTSD/S group, indicating ongoing vulnerabilities. This study also supports previous evidence that PTSD/S symptoms in HUD patients may not only stem from pre-existing trauma but also result from the addiction itself. Particularly in women without social support or stable employment, heroin addiction appears to foster a stress-reactive state similar to PTSD. Benzodiazepine misuse may serve as a proxy for inadequate coping mechanisms or an attempt at self-medicating distress. Its presence during treatment is clinically significant, given its known association with treatment failure, overdose risk, and suicidal ideation in PTSD populations [160].

### 4.6. PTSD/S and Long-Term Outcomes of Continuous Treatment

A unique longitudinal study spanning almost three decades examined seven HUD patients who received continuous OAT since the early 1990s at Santa Chiara University Hospital in Pisa, Italy [161]. These patients, initially presenting with complex dual disorders (DDs) and severe psychopathological profiles, maintained uninterrupted treatment with either methadone or buprenorphine and were systematically monitored for substance use and psychiatric comorbidities. Psychopathological assessments using the SCL-90 showed a significant reduction in symptom severity. Specifically, the W/BT dimension, initially prevalent among HUD patients, disappeared entirely in this cohort. Residual symptoms remained minimal and were probably attributable to prolonged withdrawal or hypophoric states [162]. Despite clinical remission of addiction behaviours and psychopathology, PTSD/S symptomatology persisted in five of the seven patients, as measured by the TALS instrument. These patients exhibited stress reactivity scores comparable to survivors of the 2009 L’Aquila earthquake, suggesting that chronic heroin use may itself be a traumagenic exposure [122,148].

These findings challenge the prevailing short-term model of opioid agonist treatment. Instead, they advocate viewing HUD as a chronic psychiatric illness that requires indefinite maintenance therapy when specific psychopathological markers—such as addictive behaviours, the persistence of the W/BT dimension, and H-PTSD/S symptomatology–remain unresolved. In line with existing literature [163,164], this experience supports integrated, long-term, multidimensional care strategies, particularly for patients with DDs.

### 4.7. Future Directions

The TALS, developed by the Spectrum Project, has proven valid for assessing PTSD/S symptomatology [159,165]. However, its original 116-item format is poorly suited for HUD patients, whose concentration, compliance, and tolerance for lengthy assessments are limited. In clinical practice, this has hindered its use and recruitment. A shortened version comprising 30 items (pH-PTSD/S Questionnaire) was therefore developed specifically for this population [166].

Preliminary data from the pH-PTSD/S questionnaire indicate that this trauma-spectrum reactivity is measurable and clinically significant. Originally designed to capture trauma-related symptoms in heroin-dependent patients even without discrete trauma exposure, the pH-PTSD/S reflects a functional spectrum of distress originating from the addiction experience itself [166]. In a study of individuals undergoing treatment for heroin dependence, approximately 17.4% met criteria for the pH-PTSD/S group despite not having traditional PTSD diagnoses. These individuals demonstrated significantly higher psychopathological severity, with elevated scores in depressive, somatic, anxiety, and interpersonal sensitivity dimensions, as measured by the SCL-90. They also presented a distinctive clinical profile characterised by more frequent poly-substance use, higher suicidal ideation, greater social dysfunction, and increased methadone dosage requirements. Importantly, their symptom burden closely resembled that observed in individuals with full PTSD following external traumatic events, supporting the notion that the heroin-use lifestyle itself may be a source of cumulative traumatic stress. The correlations observed between pH/PTSD/S scores and subjective wellness deficits—as measured by the DM-SWS (Deltito/Maremmani Subjective Wellness Scale) [167]—lend support to the hypothesis that heroin dependence fosters a form of latent traumatisation that persists even without external trauma.

The conceptualisation of heroin dependence as a traumatising condition—mediated by the chronic dysregulation of the endogenous opioid system and the consequent sensitisation to stress—has several important implications for future clinical practice and research.

From a diagnostic perspective, this model emphasises the need for trauma screening protocols that go beyond identifying discrete, life-threatening events. In patients with HUD, subthreshold and complex trauma symptoms should be actively evaluated using dimensional tools capable of capturing dysregulation-related phenomena. Importantly, we clarify that our hypothesis does not claim that heroin dependence, per se, fulfils Criterion A for trauma exposure as defined in the DSM-5. Instead, we propose that the chronic neurobiological and psychosocial dysregulation linked to heroin addiction may lead to PTSD-spectrum symptoms that remain clinically relevant despite not arising from a single identifiable traumatic event. The PTSD-spectrum framework may therefore provide a more accurate depiction of the emotional and cognitive impairments often seen in this population, as previously discussed [93,94].

To address potential concerns about diagnostic overlap, we differentiate the post-heroin PTSD-spectrum (pH-PTSD/S) from other clinical conditions. Unlike protracted withdrawal syndrome, which usually resolves over time and is closely associated with pharmacological cessation, pH-PTSD/S indicates a broader pattern of persistent emotional dysregulation, intrusive symptoms, and psychosocial impairment that persist independently of acute withdrawal. Similarly, while affective dysregulation is common in addiction, pH-PTSD/S is characterised by trauma-like features—such as hyperarousal, avoidance, and intrusive memories—that more closely resemble stressor-related psychopathology. In contrast to complex PTSD resulting from external adverse life events, pH-PTSD/S is hypothesised to originate mainly from the cumulative internal traumatisation linked to heroin use itself and its long-term neurobiological effects.

From a nosological perspective, we do not propose pH-PTSD/S as a formal diagnostic category within DSM-5 or ICD-11 frameworks. Instead, we view it as a dimensional construct within the broader PTSD spectrum, aimed at capturing stress-related psychopathology in individuals with HUD who do not meet full PTSD criteria but exhibit clinically significant trauma-like symptoms. This conceptualisation is not intended to inflate diagnostic categories but to support trauma-informed assessment and treatment planning through a more nuanced understanding of stress vulnerability in this population.

At the service level, the adoption of trauma-informed care models becomes crucial. The high prevalence of trauma-spectrum symptoms in HUD patients necessitates coordinated interventions that combine pharmacological stabilisation with psychotherapeutic strategies aimed at emotional regulation and stress resilience. Service organisation may also benefit from interdisciplinary models that bring together addiction specialists, trauma clinicians, and primary care providers.

From a research perspective, the trauma sensitisation hypothesis provides multiple avenues for investigation. Longitudinal studies are necessary to determine whether opioid-induced neurobiological dysregulation predicts the development or persistence of trauma-spectrum syndromes. Neuroendocrine biomarkers such as cortisol, ACTH, or CRF levels could be included in routine monitoring routines to observe individual trajectories under OAT. Additionally, neuroimaging studies might help identify structural and functional markers of opioid-induced trauma vulnerability, particularly within the amygdala, prefrontal cortex, and brainstem regions involved in stress regulation [30,99].

Finally, the development and validation of specific tools—such as the PTSD/Spectrum questionnaire proposed in this article—may help improve both diagnosis and treatment tracking in complex dual-diagnosis groups. These instruments could assist in distinguishing between traditional PTSD, subthreshold trauma syndromes, and opioid-induced stress dysregulation, thereby enabling more personalised interventions.

In conclusion, the hypothesis that heroin dependence is a traumatising condition in itself opens new perspectives for diagnosis, clinical care, and neurobiological research. Future research should aim to operationalise this model through empirical studies, clinical tools, and revised guidelines for the long-term management of HUD within a trauma-informed framework.

## 5. Implications for Trauma-Informed Care and the Role of Opioid Agonist Treatment

From a clinical perspective, this model promotes a shift from symptom-focused treatment approaches towards interventions that address the fundamental dysregulation of stress regulatory systems. In this context, opioid agonist treatment (OAT)—especially with long-acting formulations such as methadone and buprenorphine—may provide therapeutic benefits that go beyond managing withdrawal symptoms and preventing relapse. Several studies have shown that OAT can reduce hypothalamic–pituitary–adrenal (HPA) axis hyperreactivity, potentially aiding in the re-stabilisation of neuroendocrine stress responses commonly dysregulated in PTSD and HUD [44,54,65].

Furthermore, OAT may indirectly decrease PTSD-spectrum symptoms by reducing environmental instability and repeated exposure to high-risk contexts often associated with illicit opioid use. By lowering contact with traumagenic situations—such as homelessness, criminal involvement, and interpersonal violence—OAT offers a more predictable and secure psychosocial environment, which can, in turn, lower the risk of trauma re-exposure and aid emotional regulation [52,53]. These effects align with the principles of trauma-informed care, which emphasise establishing safety, predictability, and trust as the foundation for recovery.

Nonetheless, while observational data suggest that individuals receiving OAT may experience a reduction in PTSD symptom severity, the direct effects of opioid agonists on trauma-related symptoms such as intrusive recollections, dissociation, and hyperarousal remain insufficiently investigated. Randomised controlled trials are needed to clarify whether OAT helps improve PTSD symptoms independently of its effects on substance use. Additionally, the different impacts of full opioid agonists (e.g., methadone) versus partial agonists (e.g., buprenorphine) on the neurobiological mechanisms of stress regulation deserve further examination [62,86].

The converging neurobiological and psychosocial mechanisms underlying both HUD and PTSD support the perspective that OAT, when provided within a trauma-informed framework, may serve a dual purpose: reducing addiction-related behaviours and partially restoring homeostatic stress regulation. Future clinical strategies should include systematic screening for trauma-related symptoms and consider integrated pharmacological and psychotherapeutic interventions that address both substance dependence and trauma vulnerability within this highly exposed population.

## 6. Limitations and Strengths of pH-PTSD/S

This perspective article is mainly conceptual and aims to generate hypotheses. Although a consistent body of clinical research supports the proposed construct of pH-PTSD/S, it has not yet been formally validated through large-scale, multicentre studies. Many of the cited investigations are observational and conducted within specific clinical settings, which may limit the applicability of their findings to broader populations or healthcare systems. Additionally, the use of self-report instruments—although psychometrically validated—may introduce recall or reporting bias, especially among traumatised or substance-using individuals.

Several studies discussed rely on retrospective designs, which may obscure the temporal relationship between heroin use and the development of trauma-spectrum symptomatology. It is also plausible that specific neurobiological alterations attributed to heroin dependence reflect pre-existing vulnerabilities rather than addiction-related changes. Furthermore, although the dimensional model of pH-PTSD/S proposed herein offers a valuable heuristic, its integration into formal diagnostic systems remains speculative and awaits nosological refinement.

Nevertheless, this article offers a novel and clinically grounded reconceptualization of heroin addiction, not merely as a comorbid condition but as a chronic traumatising process. A key strength is the integration of empirical evidence and theoretical insights within a unified methodological and cultural framework, which improves the internal consistency of the proposed construct. By moving beyond the traditional dual disorder paradigm, this work introduces a dimensional model that captures the complex lived experiences of heroin-dependent individuals. The examination of neurobiological, psychological, and environmental mechanisms through which heroin use may generate trauma-spectrum features contributes to a more nuanced understanding of addiction psychopathology.

Finally, the perspective aligns with contemporary advances in trauma-informed and transdiagnostic approaches to psychiatric care. The inclusion of comparative analyses with traditional PTSD populations and the development of specific screening tools further improve the clinical relevance of the construct, paving the way for future research and more personalised therapeutic strategies.

## 7. Conclusions

This perspective article introduces the construct of pH-PTSD/S, proposing that heroin addiction may itself serve as a chronic traumatising condition. Rather than merely co-occurring with trauma-related disorders, pH-PTSD/S signifies a stress-spectrum syndrome intrinsically linked to the lived experience of long-term heroin dependence.

## Data Availability

Data sharing is not applicable.
